# Non-Invasive Early Molecular Detection of Gastric Cancers

**DOI:** 10.3390/cancers12102880

**Published:** 2020-10-07

**Authors:** Hiroyuki Yamamoto, Yoshiyuki Watanabe, Yoshinori Sato, Tadateru Maehata, Fumio Itoh

**Affiliations:** 1 Division of Gastroenterology and Hepatology, Department of Internal Medicine, St. Marianna University School of Medicine, Kawasaki 216-8511, Japan; ponponta@marianna-u.ac.jp (Y.W.); y2satou@marianna-u.ac.jp (Y.S.); t2maehata@marianna-u.ac.jp (T.M.); fitoh@marianna-u.ac.jp (F.I.); 2 Department of Internal Medicine, Kawasaki Rinko General Hospital, Kawasaki 210-0806, Japan

**Keywords:** liquid biopsy, DNA methylation, microRNA, long noncoding RNA, circular RNA, extracellular vesicles, gastric juice, gastric wash

## Abstract

**Simple Summary:**

This paper reviewed an update on the molecular detection of gastric cancers, focusing on various diagnostic approaches, including nonblood analytes, specifically gastric juices or washes. This comprehensive review demonstrates how liquid biopsy may be beneficial in identifying and optimizing new diagnostic approaches for gastric cancer.

**Abstract:**

Gastric cancer (GC) is a significant source of global cancer death with a high mortality rate, because the majority of patients with GC are diagnosed at a late stage, with limited therapeutic choices and poor outcomes. Therefore, development of minimally invasive or noninvasive biomarkers which are specific to GC is crucially needed. The latest advancements in the understanding of GC molecular landscapes and molecular biological methods have accelerated attempts to diagnose GC at an early stage. Body fluids, including peripheral blood, saliva, gastric juice/wash, urine, and others, can be a source of biomarkers, offering new methods for the early detection of GC. Liquid biopsy-based methods using circulating sources of cancer nucleic acids could also be considered as alternative strategies. Moreover, investigating gastric juices/washes could represent an alternative for the detection of GC via invasive biopsy. This review summarizes recently reported biomarkers based on DNA methylation, microRNA, long noncoding RNA, circular RNA, or extracellular vesicles (exosomes) for the detection of GC. Although the majority of studies have been conducted to detect these alterations in advanced-stage GC and only a few in population studies or early-stage GC, some biomarkers are potentially valuable for the development of novel approaches for an early noninvasive detection of GC.

## 1. Introduction

**Figure 1:**
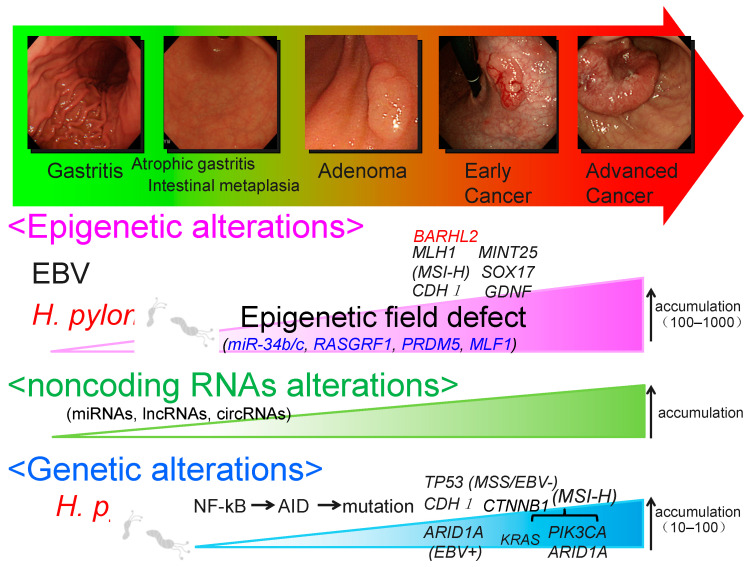
Molecular alterations in gastric carcinogenesis. The model for gastric carcinogenesis is presented based on alterations in genome, epigenome, and noncoding RNAs. Methylation of the genes in blue appears to be involved in an epigenetic field defect. *H. pylori*: *Helicobacter pylori*; MSI-H: High- frequency microsatellite instability; EBV: Epstein–Barr virus.

Gastric cancer (GC) is the fifth most frequently diagnosed cancer (one million new cases) and the third cause of yearly cancer deaths (781,000 deaths) worldwide [1]. It is a diverse cancer with various environmental etiologic factors and alternate tumorigenic pathways (Figure 1) [2,3]. One of its main etiologies is a persistent *Helicobacter pylori* (*H. pylori*) infection, but only a minor fraction of people with *H. pylori* infection develop GC [4,5]. The combination of gastroscopy with biopsy remains the standard method for the screening and diagnosis of GC [6,7].

Early detection, therapy, and precise monitoring of GC is important for improving the clinical course of patients. However, CG is mainly diagnosed using invasive methods, which sometimes have adverse events [6]. Endoscopic biopsy is the gold standard for the diagnosis of GC. The molecular analysis of specimens of advanced GC also helps to choose the therapeutic strategy. However, in certain cases, endoscopic biopsy cannot be performed, simply due to its invasiveness. Moreover, biopsy specimens do not always reflect tumor dynamics and drug sensitivities. New noninvasive biomarkers that can monitor real-time tumor dynamics are necessary for an early diagnosis, prediction of prognosis and recurrence, and evaluation of therapeutic efficacy.

Although the incidence of GC is decreasing, the survival rate remains low, mostly because many patients are asymptomatic until it reaches the late stage. Subsequently, development of noninvasive and/or minimally invasive methods for an early detection of GC is crucial to decrease GC deaths [8,9]. The newest progress in molecular characterization of GC has provided novel diagnostic and/or therapeutic approaches potentially used in clinical settings. In this review, possible clinical applications and prospects of minimally invasive and/or noninvasive biomarkers for the early detection of GC are summarized. 

## 2. Epigenetic Alteration

**Figure 2:**
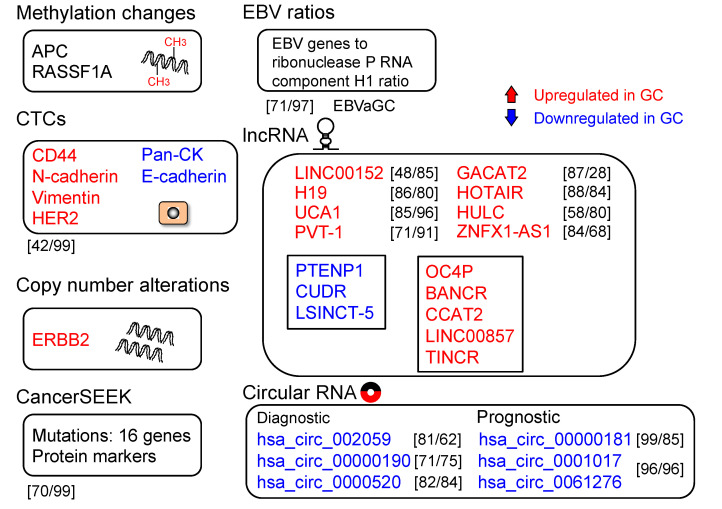
Molecular targets with the potential as diagnostic biomarkers for gastric cancer (GC). Upregulated (in red) and downregulated (in blue) markers are shown. Sensitivity and specificity are shown in square brackets.

Genomic and epigenomic modifications play important roles in GC pathogenesis [10,11,12,13]. Epigenetic regulation is necessary for the normal growth and continuation of tissue-specific gene expression patterns in mammals. Therefore, epigenetic alterations could result in impaired gene function and, eventually, malignant transformation [14]. Diverse alterations have been reported in the epigenetic mechanism of GC, including DNA methylation, nucleosome positioning, and histone modification [15,16,17,18,19]. Promoter CpG island hypermethylation results in silencing of tumor suppressor and tumor-associated genes, the most distinct epigenetic hallmark of GC. Gene hypermethylation with various biological functions has been reported in GC [15,16,17,18,19]. DNA methylation is a potent source of possible diagnostic biomarkers for GC screening [20]. Therefore, as noninvasive biomarkers of GC, DNA hypermethylation detected in body fluids, including peripheral blood, saliva, gastric wash, or urine, may be clinically important. Aberrant DNA methylation of various genes in body fluids could be a valuable biomarker for the early detection of GC (Figure 2) [17].

## 3. MicroRNAs (miRNAs)

A miRNA is a small (19–25 nucleotides) noncoding RNA sequence, which controls gene expression in the post-transcriptional phase. miRNAs are crucial for various cellular functions, such as differentiation, growth, and apoptosis [21,22,23,24,25]. Depending on the role of the target mRNA/gene, miRNAs function as tumor suppressor genes or oncogenes. Altered miRNA expression patterns in tumors are caused by various mechanisms, including genetic mutation, DNA copy number alterations, incorrect transactivation, suppression of transcription via oncogenic factors, impaired post-transcriptional process, or transcriptional silencing related to the aberrant methylation of the promoter CpG islands.

**Figure 3:**
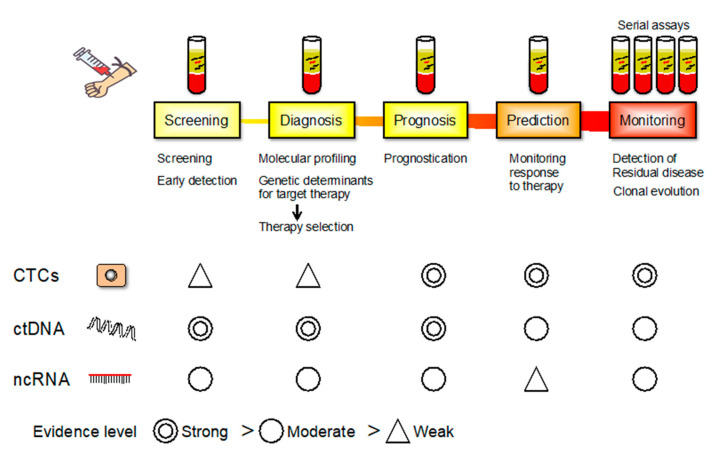
Potential applications of liquid biopsies during the course of GC management. Evidence level is shown.

Alterations in miRNA expression play a crucial role in gastric tumorigenesis [26,27,28,29]. Various miRNAs with diverse biological functions have been impaired in GC. Since tumor-derived miRNAs are present in the circulation, their levels can be quantified. Therefore, circulating miRNAs are potential diagnostic biomarkers for GC [28]. The clinical applications of altered miRNAs as noninvasive diagnostic biomarkers or therapeutic targets have certainly been described (Figure 3) [30]. miRNA isoforms are constantly expressed in a tissue- and disease-specific manner [31]. Next-generation sequencing is the most efficient technique for profiling miRNAs, resulting in the discovery of altered miRNAs with possible clinical applications.

**Figure 4:**
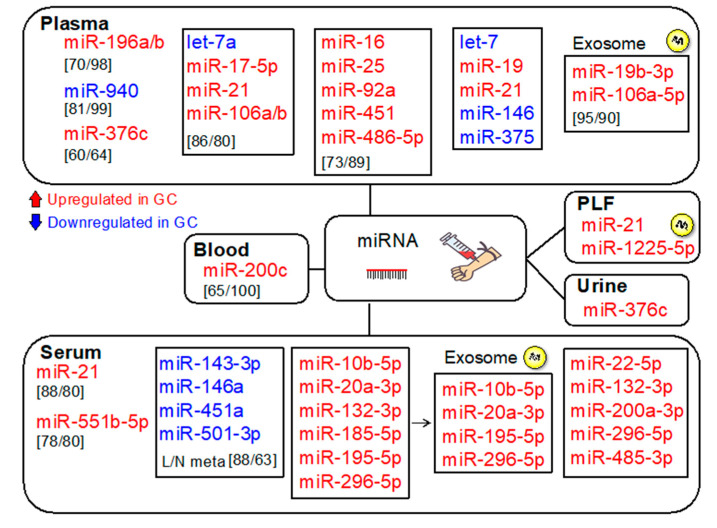
miRNAs potentially considered as diagnostic biomarkers for GC. Upregulated (in red) and downregulated (in blue) miRNAs are shown. Sensitivity and specificity are shown in square brackets. PLF: peritoneum lavage fluid.

MicroRNAs are often deregulated in a stepwise manner from chronic gastritis to preneoplastic disorders, including atrophic gastritis/intestinal metaplasia, dysplasia, and early and advanced cancer [32]. Therefore, possible diagnostic applications based on miRNA expression analysis have been reported [32]. Figure 4 summarizes the potential miRNA biomarkers for the detection of GCs.

Serum miRNA-21 levels were elevated in patients with GC, and the positive prediction rate (PPR) was 88%, whereas PPRs of CA19-9 and carcinoembryonic antigen (CEA) were 50% and 46%, respectively. The rate was 89% for stage I GC [33]. Circulating plasma miR-196a showed a higher diagnostic capability than miR-196b or combined miR-196a/b, emphasizing its ability as a potentially useful GC biomarker [34]. Plasma levels of miR-376c were also validated as a GC biomarker, even for patients with early-stage GC [35]. miR-376c levels were also elevated in the urine of patients with GC. Urinary miR-376c predicted GC with an area under the curve (AUC) of 0.70, sensitivity of 60%, and specificity of 64% [35]. Urine sample collection is easier and noninvasive compared with that of plasma [36,37]. If these results are further validated, urinary miR-376c will be a potentially valuable noninvasive biomarker for GC.

A panel of five plasma miRNAs (miR-16, miR-25, miR-92a, miR-451, and miR-486-5p) presented a high diagnostic ability for GC at the early stage (AUCs of 0.989 for the training and validation set and 0.812 for the validation set), suggesting that this panel potentially functions as a noninvasive biomarker for early-stage GC [38]. A panel of six upregulated miRNAs (miR10b-5p, miR-20a3p, miR132-3p, miR185-5p, miR195-5p, and miR296-5p) showed diagnostic accuracy for GC (AUCs of 0.764 for the training set and 0.702 for the validation set) [39]. Among them, miR10b-5p, miR20a-3p, miR195-5p, and miR296-5p were also elevated in serum exosomes of patients with GC. Importantly, not all circulating miRNAs are released from exosomes [40]. Argonaute2 complexes reportedly transport a subset of circulating miRNAs, irrespective of vesicles in the plasma [41].

A panel of five upregulated serum miRNAs (miR-22-5p, miR-132-3p, miR-200a-3p, miR-296-5p, and miR-485-3p) showed diagnostic accuracy for cardia GC (AUCs of 0.766 for the training set and 0.724 for the validation set) [42]. Exosomal miR-19b-3p, miR-106a-5p, and their combination discriminated GCs from non-GCs with AUCs of 0.769, 0.786, and 0.814, respectively. Moreover, this combination distinguished GC from non-GC with 95% sensitivity and 90% specificity in the validation set [43]. miR-21 and miR-19 have been shown to increase and let-7, miR-146, and miR-375 decrease in *H. pylori*-infected GC patients, suggesting the potential of these miRNAs as biomarkers for the early detection of GC [44].

Recent findings suggest the role of miRNAs in the regulation of gastric cancer stem cells (GCSCs). A better understanding on molecular mechanisms and target genes involved in the GCSC regulation could accelerate the development of new strategies in the early detection of GC [45].

## 4. Epigenetic Field Defects and miR-34b/c Methylation 

Aberrant CpG island hypermethylation is often associated with miRNA silencing. MicroRNA-34b/c methylation was frequently detected in GCs (83/118, 70%) [46]. Moreover, methylation levels in the gastric mucosa of patients with multiple GCs were higher than in those of patients with single GC or non-GC individuals with *H. pylori* infection. These findings suggest that miR-34b/c methylation is implicated in a gastric epigenetic field defect and could be a promising biomarker to analyze the risk of multiple GCs.

After an endoscopic resection of a primary GC, a secondary GC could develop metachronously. However, predicting the development of metachronous GC based on clinicopathological features of primary GC alone is difficult. DNA hypermethylation in the nontumor gastric mucosa is involved in tumorigenesis and could be a valuable biomarker for GC risk. In a prospective study of 129 patients with early GC after the curative endoscopic resection, metachronous GCs occurred in 17 patients (13%) [47]. Multivariate analysis indicated that miR-34b/c methylation in the gastric body is an independent predictive marker for the occurrence of metachronous GC, suggesting its clinical application.

## 5. Circulating Tumor Cells (CTCs) 

**Figure 5:**
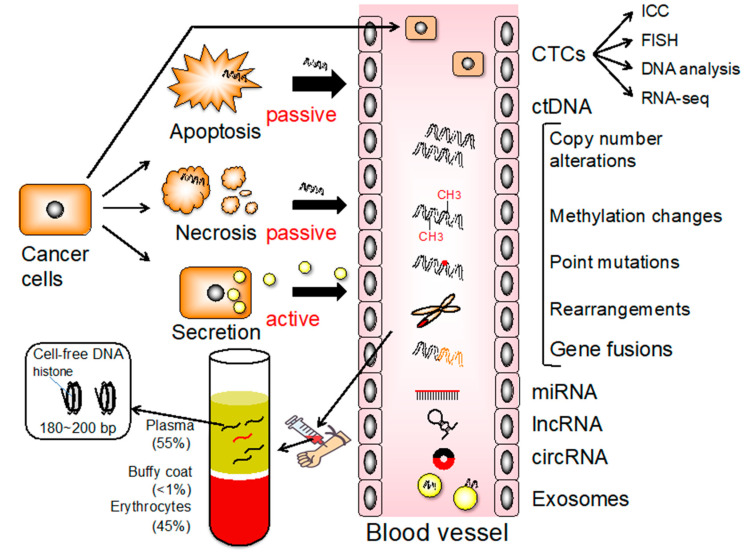
Origins and range of alterations in liquid biopsies. Left bottom, cell-free DNA (cfDNA) predominantly consists of nucleosome-protected DNA shed into the bloodstream by cells undergoing apoptosis.

Circulating tumor cells are a heterogeneous population of cells originating from primary tumors that enter the bloodstream, representing an essential step in hematogenous metastasis (Figure 5). As a liquid biopsy, CTCs have received considerable attention because they are easily accessible in the peripheral blood and can also be used to monitor tumor dynamics (Figure 3) [48]. Due to CTC heterogeneity, various methodologies have been developed to isolate and count them based on specific molecular or phenotypic characteristics. Detection of CTC is potentially useful for evaluating tumor dynamics and for monitoring GC treatment responses (Figure 3) [49].

The gastric tissue adjacent to the tumor reportedly shows an aberrant gene signature and biological characters, suggesting molecular characteristics partly different from both GC and healthy tissues. Therefore, using the gastric tissue adjacent to the tumor as a healthy control tissue in the analysis could lead to inappropriate results. Using circulating sources of biomarkers may be less laborious and misleading. Conversely, the methodology using a differential gene expression among normal, adjacent, and tumor tissues could help to specifically identify cancer biomarkers that could be used as targets on CTCs [50].

According to a meta-analysis of the diagnostic accuracy of diverse CTC detection procedures, the combined sensitivity and specificity of CTCs for GC were 42% and 99%, respectively (Figure 2) [51]. Epithelial cell adhesion molecule (EpCAM) is a CTC marker in various cancer types, whereas cluster of differentiation 44 (CD44) is a GCSC marker. The sensitivity and specificity of EpCAM+CD44+ cell detection to identify patients with GC were 92.3% and 100%, respectively, suggesting that CD44+ CTCs are a candidate biomarker for the detection of GC [52]. Despite the fact that challenges in techniques remain, CTCs are still potential biomarkers for the detection of GC [53].

## 6. Cell-Free DNA (cfDNA)

Circulating tumor DNA (ctDNA) has been identified as a potentially useful biomarker for the early detection of cancer, prediction of prognosis, detection of therapeutic targets, and real-time monitoring of tumor dynamics (Figure 3). Deriving from primary and/or metastatic tumors, ctDNA can be used for various analyses, such as genetic mutations, rearrangements, copy number variations, and methylation status (Figure 5) [54]. Various cancer-specific genetic and/or epigenetic alterations have been examined as possible ctDNA-based biomarkers for the early detection of GC [20]. Tumor-specific SNVs and gene amplifications can be detected in the plasma of a majority of patients with GC (Figure 2) [55]. Analyzing the copy number variation is more challenging due to the short length and uneven distribution of ctDNAs [54].

In general, ctDNA represents a fraction of cfDNA that is substantially increased in late-stage tumors [56]. However, ctDNA could be detected in patients with tumors at an early stage [57,58]. Methylation of the promoter of various genes in ctDNA has been frequently detected in patients with early-stage GC (Figure 2). CancerSEEK is a unique, multianalyte blood test that concurrently analyzes mutations and cancer-related protein biomarkers. Circulating tumor DNA-based multiplex polymerase chain reaction (PCR) analysis can detect mutations at 2001 genomic loci of 16 genes, whereas protein biomarker levels are analyzed using immunoassays. The sensitivity and specificity for GC were 70% and >99%, respectively, with only 7 out of 812 control individuals being positive [59]. This assay combines ctDNA and protein biomarkers to increase the diagnostic accuracy and support in the identification of specific tumors, including GC [60].

Moreover, cfDNA could reportedly recognize Epstein–Barr virus (EBV)-associated gastric carcinoma (EBVaGC) and monitor cancer dynamics [61]. Using EBV genes-to-ribonuclease P RNA component H1 ratios (EBV ratios) analyzed using quantitative real-time PCR, cell-free EBV DNA was found in patients with GC with a sensitivity of 71% and specificity of 97%. The plasma EBV ratios in patients with EBVaGC decreased after treatment and increased during tumor progression/recurrence. The plasma EBV ratio appears to be useful for the recognition of EBVaGC and/or for real-time monitoring of tumor dynamics. Analysis of ctDNA is possibly a sensitive procedure for the detection of low-frequency mutations; however, further studies are necessary to verify if and how the analyses could be used in a clinical setting [55].

## 7. Long Noncoding RNAs (LncRNAs)

Long noncoding RNAs are long (>200 nucleotides) transcripts with no or restricted ability to encode proteins. They regulate various biological processes, such as transcription, splicing, translation, and function as molecular sponges for miRNAs [62]. Long noncoding RNAs are highly stable and circulate in body fluids, and their plasma levels were reportedly correlated with tumor tissue levels. Altered lncRNAs could be used to detect early-stage cancer and to predict prognosis, risk of metastasis, and recurrence [63,64]. Currently, >56,000 human lncRNAs have been recognized, and various lncRNAs have been dysregulated in GC [65,66].

Various lncRNAs that are highly expressed in GC tissues have been analyzed as GC biomarkers [67] (Figure 2). Expression of plasmacytoma variant translocation 1 (PVT1) in GC tissues discriminated GC from non-GCs with an AUC of 0.728, sensitivity of 80%, and specificity of 60%. PVT1 levels in gastric juice (GJ) were higher in GCs than that in non-GCs [68]. Highly upregulated in liver cancer (HULC) lncRNA is involved in gastric tumorigenesis. Overexpression of HULC lncRNA in GC cell lines enhanced the growth and invasion, whereas it suppressed apoptosis and induced autophagy. The epithelial–mesenchymal transition (EMT) phenotype was reversed by HULC silencing [69]. The plasma HULC and ZNFX1-AS1 levels were higher in GCs than that in non-GCs. The HULC levels discriminated GCs from non-GCs with an AUC of 0.65, sensitivity of 58%, and specificity of 80%. Levels of ZNFX1-AS1 discriminated GCs from non-GCs with an AUC of 0.85, sensitivity of 84%, and specificity of 68% [70]. Plasma AA174084 levels significantly decreased in GCs postoperatively and were related to the depth of invasion and lymph node metastasis [71].

Long noncoding RNA H19 reportedly enhances GC cell growth and suppresses apoptosis [72]. Plasma H19 levels were higher in GCs than that in non-GCs [73,74,75] and discriminated between early-stage GCs and non-GCs with an AUC of 0.877, sensitivity of 86%, and specificity of 80% [73]. In addition, plasma H19 levels were lower in patients with GC postoperatively than preoperatively [73]. Therefore, the plasma H19 level could be a biomarker for the detection of early-stage GC. Plasma levels of long intergenic nonprotein-coding RNA 152 (LINC00152) were higher in GCs than those in non-GCs, and levels were higher in patients with GC postoperatively than preoperatively [76]. One of the possible mechanisms for its stability in the blood is thought to be protection by exosomes [76].

The levels of plasma gastric cancer associated transcript 2 (GACAT2) were higher in patients with GC than in non-GC controls, as well as in preoperative than that in postoperative samples. Relative changes in GACAT2 levels postoperatively were related to perineural invasion and to the lymph node and distal metastasis [77]. Plasma GACAT2 levels discriminated between GC and non-GC groups with an AUC of 0.622, sensitivity of 87%, and specificity of 28% [77]. Human urothelial carcinoma-associated 1 (UCA1) is involved in gastric tumorigenesis and is increased in patients with GC [78]. Hox transcript antisense intergenic RNA (HOTAIR) is involved in gastric tumorigenesis [79], and it reportedly enhances GC cell growth and suppresses apoptosis [70]. Moreover, enhanced HOTAIR expression was related to higher grades, metastasis, and advanced tumor stages. Plasma HOTAIR levels were higher in GCs than in non-GCs [79].

Genome-wide lncRNA microarray profiling identified a panel of five plasma lncRNAs (AOC4P, BANCR, CCAT2, LINC00857, and TINCR) that distinguished GCs from non-GCs. The lncRNA-based index was significantly diminished at 14 days postoperatively, showing its potential for monitoring tumor dynamics [80]. Similarly, a panel of three serum lncRNAs (PTENP1, CUDR, and LSINCT-5) reportedly distinguished early GC from non-GC with AUCs of 0.920 and 0.829 for the two sets of samples [81]. Additional analyses of lncRNAs are necessary to further support their clinical application.

## 8. Circular RNAs (CircRNAs)

Circular RNA is a noncoding RNA that forms a closed loop lacking 5’ and 3’ ends [82]. Circular RNAs are generated from introns or exons through loop introns or reverse splicing. The most common type is the exonic type. Circular RNAs are also generated from nonlinearly reverse spliced exon transcripts from pre-mRNA [83], including one or more exon loops and exon–intron hybrid loops (EIcircRNA). The intron also circularizes and creates circRNAs (ciRNAs).

Circular RNAs regulate gene expression via interactions with miRNAs [84]. Altered circRNAs are related to tumorigenesis, including GC [85]. Altered circRNA–miRNA–mRNA interactions are also implicated in GC. Various up- or downregulated circRNAs have been reported in GC tissues [86]. Because of their abundance, stability, tissue-specific expression, and broad circulation in diverse body fluids and exosomes, circRNAs could be new diagnostic biomarkers and GC therapeutic targets [87]. CircRNAs derived from exosomes (ex-circRNAs) could be a new diagnostic and/or prognostic marker.

Altered circRNA levels in body fluids have been reported to be comparable to those in tumor tissues and are considered possible biomarkers (Figure 2). Plasma hsa_circ_0000520 levels showed a better diagnostic accuracy than tissue levels. The AUC, sensitivity, and specificity of plasma hsa_circ_0000520 were 0.897, 82%, and 84%, respectively, which were higher than those of the tissue levels (0.613, 54%, and 86%, respectively) [88]. Plasma hsa_circ_002059 and hsa_circ_0000190 levels could be diagnostic biomarkers for GC [85,89]. Other circRNAs, such as hsa_circ_00000181, hsa_circ_0001017, and hsa_circ_0061276, could be prognostic biomarkers for GC, with high sensitivity and specificity [90,91]. Although circRNAs can be considered as potential GC biomarkers, their usefulness requires further validation using large cohorts.

## 9. Extracellular Vesicles (EVs)

Extracellular vesicles are membrane-encased structures released from various cells [92,93]. They include exosomes, microvesicles, and apoptotic bodies and are classified into three major molecules according to particle size and biogenesis processes. Exosomes are 30–200 nm in size and are generated as intraluminal vesicles inside the lumen of multivesicular bodies. Microvesicles are 100–1000 nm in size and are generated when the cellular membrane is partly pinched off and directly discharged from originating cells. The apoptotic body is 500–2000 nm in size and is generated in the late phase of apoptosis. Plasma cfDNA is derived from cells that undergo necrosis or apoptosis, which might not reflect viable tumor cells. Conversely, exosomal nucleic acids (exoNAs), including DNAs and RNAs, are actively released from viable tumor cells and, therefore, could better reflect tumor dynamics (Figure 5) [94]. Moreover, exosomes can protect nucleic acids from degradation [95,96,97,98].

Cancer-related EVs play important roles in creating advantageous microenvironments for tumor cells through the interaction with diverse neighboring or distant cells. Extracellular vesicles stimulate EMT, angiogenesis, and immunosuppression through the transportation of functional molecules, such as nucleic acids, metabolites, and proteins. Extracellular vesicles also transfer cancer-related antigens to antigen-presenting cells. As EV cargoes derived from tumors hold biomolecular characteristics of their sources and tumor cells dynamically secrete EVs into body fluids, EVs are thought to be potentially promising resources for the identification of biomarkers.

Extracellular vesicles hold molecular cargoes that are protected from degradation in body fluids [99,100]. Cell-free DNAs are usually detected as short fragments (<100 bp) because they are severely degraded in the blood [100]. Conversely, DNA fragments (100 bp to 17 kb) in EVs represent a broader range of molecular information that could be detected in EV-DNAs [101]. Extracellular vesicle cargoes directly reflect the properties of their sources. For instance, BRCA2, NOTCH1, TP53, and KRAS gene mutations were detectable in EVs from patients with pancreatic and ampullary cancer [102]. Mutations of KRAS were detected in exoDNA in patients with early-stage pancreatic cancer [103]. In lung cancer, driver mutations were detectable in exoDNA more sensitively than those in cfDNA [104]. Cancer-related EVs also contain tumor-specific protein cargoes, suggesting that EVs can be considered as new diagnostic cancer biomarkers.

## 10. Analysis of Gastric Juices/Washes 

### 10.1. Gastric Juice (GJ)-Based Biomarkers

**Figure 6:**
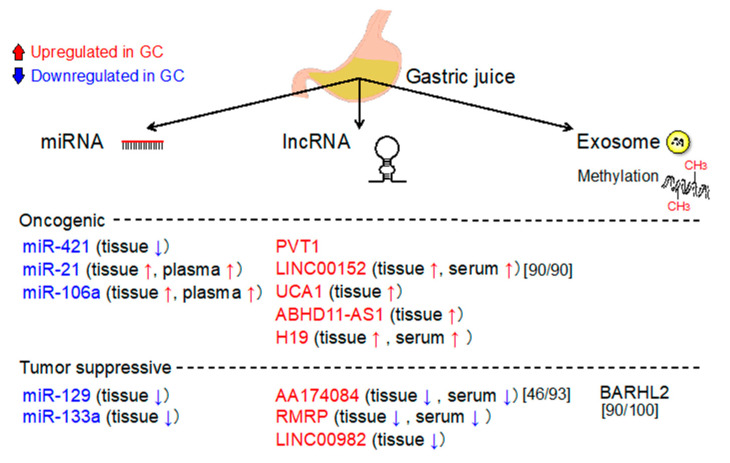
Gastric juice (GJ)-based molecular targets potentially considered as diagnostic biomarkers for GC. Upregulated (in red) and downregulated (in blue) markers are shown. Sensitivity and specificity are shown in square brackets.

Gastric juice could be a good biomarker source for GC, as it is directly secreted from cells without elimination by the liver [105]. Altered expression levels of oncomiRs (miR-421, miR-21, and miR-106a) and tumor suppressor miRNAs (miR-129 and miR-133a) have been reported in GJ samples from patients with GC than those from without GC (Figure 6). These GJ miRs are potential biomarkers for the detection of GC. As a possible mechanism underlying the decreased and increased oncomir levels in GJ and tissue/serum/plasma, respectively, GC cells may diminish the export of oncogenic miRs into GJ and enhance their release into the extracellular environment [106]. Thus, miRNA alteration in GJ could represent an alternative for the detection of GC via biopsy. 

With regard to lncRNAs, the GJ AA174084 levels were higher in GCs than that in non-GCs [69]. Gastric juice AA174084 levels discriminated between GC and non-GC groups with an AUC of 0.848, sensitivity of 46%, and specificity of 93%. The AUC for GJ was higher than that for the tissue level (0.676). Similarly, LINC00152 discriminated GCs from non-GCs with an AUC of 0.645, sensitivity of 90%, and specificity of 90% [107]. Gastric juice PVT1 levels were significantly higher in GCs than in non-GCs, suggesting that PVT1 could be a useful biomarker for the early detection of GC [71]. Gastric juice ABHD11-AS levels were higher in patients with GC and were related to clinicopathological characteristics [108]. More importantly, the PPR for early GC was 71%.

### 10.2. DNA Methylation Analysis Using Gastric Washes (GWs)

**Figure 7:**
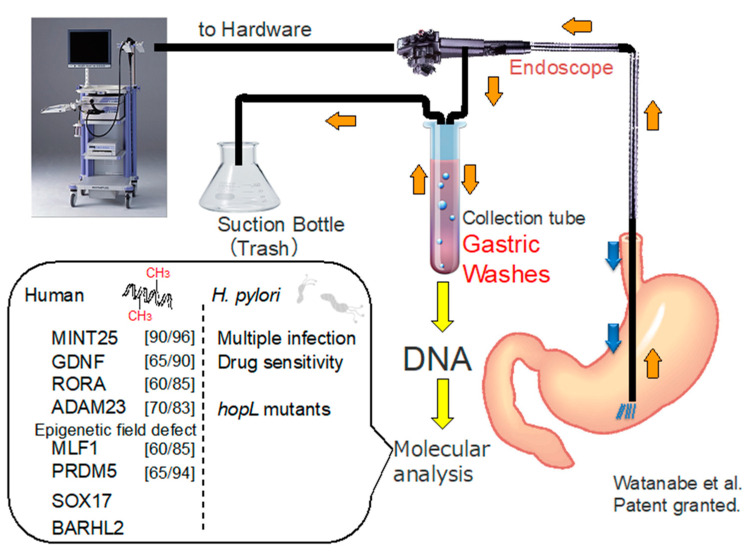
Gastric wash (GW)-based molecules with the potential as diagnostic biomarkers for GC. Sensitivity and specificity are shown in square brackets.

Since abundant mucosal cells could be obtained in GJ, DNA biomarkers using GJ were a potential method to detect GC. However, using GJ as a DNA biomarker has been unfeasible, as DNA is easily degraded due to gastric acidity. Thus, GWs are an alternative source for the detection of DNA alterations (Figure 7). A novel method for the detection of GC using GW DNA methylation analysis has been developed [109]. DNA methylation levels of MINT25 and GDNF were the most sensitive markers for GCs at the early stage, whereas methylation levels of MLF1 and PRDM5 were markers for a field defect in the stomach. The methylation levels in GWs were closely correlated with those in tumor biopsy samples. Moreover, MINT25 methylation in GW discriminated GC from non-GC with an AUC of 0.961, sensitivity of 90%, and specificity of 96%, suggesting that it is a potentially sensitive and specific biomarker for the detection of GC. 

Although endoscopic therapy is generally used for GC at the early stage, predictive markers for residual cancer and/or recurrence after an endoscopic resection are necessary. Gastric wash DNA-based methylation analysis of Sox17 has been shown to be helpful for an early diagnosis of recurrence after an endoscopic treatment in patients with GC [110].

### 10.3. Gastric Wash or Gastric Juice Exosomal DNA-Based Methylation Analysis of BARHL2

Increased methylation levels of BARHL2 were detected in GW-derived DNA in patients with early GC before endoscopic therapy, whereas methylation levels were considerably reduced, following a curative endoscopic therapy. These results indicate that BARHL2 methylation could be helpful for the diagnosis of residual cancer after an endoscopic resection and potentially prediction of cancer recurrence [111]. Moreover, BARHL2 methylation in GJ-derived exoDNAs discriminated GC from non-GC with an AUC of 0.923, sensitivity of 90%, and specificity of 100%. Therefore, GW or GJ exoDNA-based methylation analysis of BARH2 could be a promising biomarker for the early detection of GC.

### 10.4. Analysis of Helicobacter pylori Genotypes Using GWs

*H. pylori* is an important factor in the occurrence of GC. Eradication of *H. pylori* significantly reduces the occurrences of GC. The effectiveness of GW-based analysis of drug resistance in *H. pylori* has been shown [112]. Moreover, a quantitative pyrosequencing analysis using GWs was developed to evaluate the diversity and abundance of *H. pylori* genotypes [113]. These results obtained using GWs were comparable to those obtained using biopsy samples. Moreover, GW-based quantitative pyrosequencing revealed that *H. pylori* mutant strains were enriched after an eradication therapy [114]. This approach could be useful to analyze antibiotic resistance in *H. pylori* and to evaluate the potential risk of GC. Recently, GC-related genetic variants of *H. pylori* strains were determined using GW-based whole genome analysis with a single-molecule real-time technology. These results suggest that the *hopL* variant is related with the GC development and could be a genetic biomarker of *H. pylori* virulence and risk of GC [115].

## 11. Ongoing Studies Concerning the Early Molecular Detection of GC

Ongoing studies (prospective observational or retrospective) concerning the early molecular detection of GC are summarized (Table S1). With regard to GW, we have done a multicenter prospective cohort study with >400 patients with early GC who had undergone endoscopic resection to determine whether GW-based DNA methylation levels could predict the risk of developing metachronous GCs. Data analysis of this study is underway.

## 12. Conclusions

Recent progress in the biomolecular characterization of GC has delivered potentially novel clinical diagnostic and/or therapeutic approaches. Molecular analyses based on noninvasively obtained body fluids, including GJ/GW, are potentially useful for the early detection of GC. However, population studies or studies on early-stage GC to determine its usefulness as an early detection screening tool are still limited. Although validation using independent cohorts in prospective studies is necessary, body-fluids-based molecular testing could be a new noninvasive diagnostic biomarker with high accuracy for the early detection of GC and may be incorporated into GC clinical settings in the near future.

## Supplementary Materials

The following are available online at https://www.mdpi.com/2072-6694/12/10/2880/s1, Table S1: Ongoing studies concerning the early molecular detection of GC.
